# Valorization of Oil Cakes in Two-Pot Lactone Biosynthesis Process

**DOI:** 10.3390/foods14020187

**Published:** 2025-01-09

**Authors:** Jolanta Małajowicz, Agata Fabiszewska, Bartłomiej Zieniuk, Joanna Bryś, Mariola Kozłowska, Katarzyna Marciniak-Lukasiak

**Affiliations:** 1 Department of Chemistry, Institute of Food Sciences, Warsaw University of Life Sciences, Str. Nowoursynowska 159C, 02-776 Warsaw, Poland; agata_fabiszewska@sggw.edu.pl (A.F.); bartlomiej_zieniuk@sggw.edu.pl (B.Z.); joanna_brys@sggw.edu.pl (J.B.); mariola_kozlowska@sggw.edu.pl (M.K.); 2 Department of Food Technology and Assessment, Institute of Food Sciences, Warsaw University of Life Sciences, Str. Nowoursynowska 159C, 02-776 Warsaw, Poland; katarzyna_marciniak_lukasiak@sggw.edu.pl

**Keywords:** biotransformation, waste raw materials, hydroxy fatty acids, γ-dodecalactone, lactic acid bacteria, *Yarrowia lipolytica*

## Abstract

Oil cakes are biomass wastes created by pressing oil from oilseeds. Their chemical composition (including high fat or protein content, a favorable fatty acid profile, and a high proportion of unsaturated acids) makes them valuable raw materials not only in animal feeding but are increasingly gaining popularity in biotechnological processes. This article examines the possibility of valorizing oil cakes using the lipid fraction extracted from them or their raw form in a two-pot biosynthesis process of GDDL—a cyclic ester with a creamy-peach aroma. This study tested five types of oil cakes (hemp seeds, rapeseed, safflower, camelina, and flax), analyzing their physicochemical composition and the fatty acid profile of their lipid fraction. Due to the high content of oleic acid (over 62% lipid fraction) and the wide availability, rapeseed cake was used in the biotransformation process. The synthesis of GDDL involved a three-step process: hydrolysis of triacylglycerols, hydration of oleic acid (via lactic acid bacteria in anaerobic conditions), and β-oxidation (via *Yarrowia* yeast, aerobic process). The analysis showed that it is possible to produce because of the two-pot biotransformation of approximately 1.7 g of GDDL/dm^3^. These results highlight the process’s potential and justify the feasibility of waste valorization. The proposed biotransformation requires optimization and is a good example of the application of the circular economy in food processing and waste management.

## 1. Introduction

Lactones are cyclic esters formed by esterification between the hydroxyl group and the carboxyl group of a hydroxy fatty acid [1]. Lactones with five- (γ) and six- (δ) membered rings are the most diverse, chemically stable, and commercially appreciated by the food and perfume industries [2,3]. These compounds are key flavors to impart sweet, peachy, and/or apricot, coconut-like, fruity, creamy, buttery, and floral notes to food formulations [4,5]. Lactones from the groups mentioned above can be obtained in three ways: by extraction from natural sources, chemical synthesis, or biotransformation of hydroxy fatty acids. Obtaining lactones on an industrial scale by extraction from natural raw materials is relatively expensive due to their low concentration and the complex purification process. The commonly used chemical synthesis based on the oxidation of cyclic ketones with hydrogen peroxide in the presence of a strong acid catalyst raises environmental concerns. This is due to the high consumption of solvents and acids, the generation of racemic mixtures as reaction products, and the fact that such syntheses are currently not well accepted by consumers [6]. For this reason, biotechnological methods based on the processes of degradation of hydroxy fatty acids (HFA) [7,8,9], reduction in unsaturated lactones [10], and α, ω-oxidation of fatty acids or alkanes [11] have gained popularity.

Most of the attention in the literature has been devoted to the biosynthesis of natural lactones via the biotransformation of hydroxylated fatty acids using oleaginous yeast or fungi [12,13,14]. An example is the synthesis of gamma-decalactone based on the biotransformation of castor oil (the main component of which is ricinoleic acid—10-hydroxy-12-(*Z*)-octadecenoic acid) via the yeast *Yarrowia lipolytica* [15,16,17]. A significant limitation associated with the production of lactones via biotransformation of HFA is the availability of the substrates themselves. HFA occur in nature—they are extracted from plants but are not a commonly occurring substrate. Only ricinoleic acid (constituting almost 90% of castor oil triglycerides) is important on an industrial scale [18]. However, castor oil, from which the acid is extracted, has high toxicity related to the extraction procedure. The seeds of the *R. communis* castor plant (from which the oil is extracted) contain ricin—a potent toxin that enters the body, among others, due to inhalation [19].

Considering current consumer trends, such as environmental protection and sustainable development, alternative paths for lactone synthesis are currently being sought, using more accessible substrates from waste raw materials for valorization. Consumers are more environmentally conscious than ever, and the food industry is increasingly adopting a circular approach to sustainable development. The aim is for resources of the highest possible quality to be recovered, reused, and used for as long as possible.

Research is being conducted to develop biocatalytic methods based on the hydration of the double bond of unsaturated fatty acids, leading to the formation of HFA. The conversion of unsaturated fatty acids into HFAs is possible via oleate hydratase enzymes (EC 4.2.1.53). These enzymes selectively introduce a hydroxy group to the position 10 or 13 (10-hydratases or 13-hydratases, respectively) of the unsaturated fatty acid chains [20,21,22,23]. Marella et al. [24] successfully synthesized γ-dodecalactone (GDDL) and δ-decalactone from non-hydroxylated fatty acids by genetically modifying the *Yarrowia lipolytica* strain. Serra et al. described the natural activity of hydratases in probiotic microorganisms [25,26].

This article describes the research on the synthesis of lactones via mixed cultures using waste materials from the oil industry. The selected material for this study was oil cakes obtained from the OleoVital company (Poland) after the cold-pressing of oil from oil seed plants. The oil cakes from hemp seeds, rapeseed, safflower, camelina, and flax were analyzed. The research work was divided into two stages, one of which concerned the characterization of the waste material in terms of its usefulness in the synthesis of lactone (including, among others, the assessment of the content of unsaturated fatty acids, which are a precursor of biotransformation), and the second stage involved the biosynthesis of cyclic ester with a creamy-peach aroma itself. The research aimed to develop a two-pot biosynthesis process of γ-dodecalactone from waste raw materials of the fat industry via the lactic acid bacteria *Lactobacillus plantarum* and the oleaginous yeast *Yarrowia lipolytica*.

## 2. Materials and Methods

### 2.1. Materials

The research material consisted of oil cakes, the residue obtained after pressing oil from hemp seeds, rapeseed, safflower, camelina, and flax, using the cold method at a temperature not exceeding 40 °C. The oil cakes came from the OleoVital Company, (Grajów, Poland). All chemicals used were of analytical grade and were purchased from Sigma-Aldrich (Saint Louis, MO, USA), Merck Life Science Sp.z.o.o. (Poznań, Poland), or Avantor Performance Materials S.A. (Gliwice, Poland). The components of the microbiological media were purchased from BTL (Łódź, Poland).

### 2.2. Methods

#### 2.2.1. Methods Used in the Analysis of Oil Cakes

##### Determination of Dry Mass

Dry matter in the oil cakes was determined by the conventional method. On the RADWAG PS 3500 scale, 5 g of each cake was weighed in weighing vessels with an accuracy of 0.001 g, then dried in a KBC-65 G dryer at 100 °C for at least 24 h. After drying and cooling in a desiccator, the contents of the vessels were weighed again, and the dry matter content was calculated based on the loss of mass. The determination was performed in three repetitions.

##### Oil Extraction

Approximately 15 g of oil cakes were weighed (with an accuracy of 0.001 g) and then crushed in a mortar. The crushed raw material was transferred to a thimble and placed in a Soxhlet apparatus. Approximately 1.5 h of extraction (10 extraction cycles) was carried out using a portion of 150 mL of hexane. After extraction, the solvent was evaporated (Büchi B-490 Heating Bath evaporator, Büchi, Flawil, Switzerland), the flasks with the extracted oil were weighed, and the efficiency of the extraction process was calculated using Equation (1). The determination was performed in triplicate.

Oil yield (wt%) = Mass of extracted oil/mass of oil cakes (1)

##### Protein Determination

The total protein content of the oil cakes was ascertained using the Kjeldahl method (AOAC, 2002) [27]. A Tecator Digestion System (Hilleroed, Denmark) and a Tecator Distillation unit, Kjeltec 1026, were used for the analysis. The sample size used in the Kjeldahl procedure was around 1 g. Samples were weighed and transferred into a Kjeldahl digestion (System 6, 1007 Digester, Shandong, China) flask containing 7 g of catalyst (prepared by mixing K_2_SO_4_ and CuSO_4_ × 5 H_2_O) and 12 mL of concentrated H_2_SO_4_. The digestion temperature was 420 °C, and the duration was 1 h. The sample was cooled to room temperature after digestion using electric heat and vapor extraction. By distillation, ammonium hydroxide was trapped as ammonium borate in a boric acid solution (mass concentration c = 40 g/L). Total nitrogen was determined by titration with standardized HCl to a mixed indicator endpoint (1 mg/mL bromocresol green and 1 mg/mL methyl red in ethanol of volume concentration r = 950 mL/L). The determination was performed in triplicate. Equations (2) and (3) were used for calculations:

Kjeldahl nitrogen (%) = ((VS − VB) × M × 14.01)/(W × 10),(2)

and

Crude protein (%) = % Kjeldahl N × F(3)

where VS = volume (mL) of standardized acid used to titrate a test; VB = volume (mL) of standardized acid used to titrate reagent blank; M = molarity of standard HCl; 14.01 = atomic weight of N; W = weight (g) of test portion or standard; 10 = factor to convert mg/g to percent; and F = factor to convert N to protein—5.3.

##### Determination of Acid Value

The acid value was determined using potentiometric titration. Approximately 1 g of oil extracted from individual cakes was weighed (Soxhlet method extraction using dichloromethane as an extractant), then dissolved in 70 mL of a 1:1 (*v*/*v*) ethanol–diethyl ether mixture and automatically titrated using a TitraLab AT1000 Series titrator, HACH LANGE Sp. z o.o., Wrocław, Poland. The determination consisted of neutralizing free fatty acids in the tested oil titrated with a 0.1 molar potassium hydroxide solution against the phenolphthalein indicator until a light pink color was obtained, lasting 30 s. The result was expressed in mg KOH/g oil. The determination was performed in triplicate.

##### Fatty Acids Composition Study

The analysis of the fatty acid composition was carried out using the gas chromatography method. A GC YL6100 chromatograph was used with a flame ionization detector (FID) and a BPX70 capillary column with an internal diameter of 0.25 mm × length of 60 m and a film thickness of 0.25 μm. For analysis, fatty acids were converted into volatile derivatives by esterification with methanol, per the PN-EN ISO 5509:2001 standard [28]. The fatty acid methyl esters separation was performed under the following conditions: The initial temperature was 60 °C and held for 5 min. Then the temperature was increased at a rate of 10 °C/min to 180 °C and then increased at a rate of 3 °C per minute to 230 °C. At this temperature, a hold was performed for 15 min. The injector and detector temperatures were 225 °C and 250 °C, respectively. Nitrogen was used as the carrier gas. Fatty acids were identified based on retention time compared with standards.

##### Determination of the Oxidative Induction Time

Pressure Differential Scanning Calorimetry (PDSC) was used to define the oxidative stability of oil from oil cakes. To determine the induction time for the oxidation reaction of oil, experiments were carried out with the help of a DSC Q20 apparatus (TA Instruments, New Castle, DE, USA) linked to a high-pressure chamber. The weight of the oil used in the test ranged from 3 to 4 mg. Oil samples were placed in small aluminum pans in the oxygen atmosphere and under a pressure of 1400 kPa. Measurements were taken isothermally at 120 °C. Oxidation induction time was determined from PDSC curves. The result of the PDSC measurement was expressed in minutes.

##### Total Phenolic Content Analysis

The total polyphenol content in the oil was evaluated using the Folin–Ciocalteu method based on spectrophotometric measurement. To prepare samples for analysis, 1 g of each oil cake oil was dissolved in 5 mL of n-hexane and extracted with 5 mL of methanol (99.8%; Avantor, Gliwice, Poland) at an ambient temperature on an IKA shaker for 10 min. After centrifugation (MPW-352 centrifuge—5 min, 6000 rpm), the methanolic layer was separated from the lipid phase. An amount of 0.18 mL of the methanolic fraction was placed in tubes, and 4.92 mL of distilled water was added. To the prepared solution, 0.3 mL of Folin–Ciocalteu reagent (Merck Life Science, Poznań, Poland) was added, 3 min waited, and then 0.6 mL of supersaturated sodium carbonate (CHEMPUR, Piekary Śląskie, Poland) was added. The samples were incubated for 1 h without access to light at 25 °C. The absorbance of the solutions was measured using a spectrophotometer (Spektrofotometr RayLeigh UV—1601, EnviSense, Lubin, Poland) at a wavelength of 750 nm against a blank sample. The content of polyphenols was calculated as gallic acid equivalent (mg GAE/100 g). The analysis was conducted three times.

##### Statistical Analysis

The statistical analysis was performed using Statistica version 13.3 software. (StatSoft, Krakow, Poland). Analysis of variance (ANOVA) and Tukey’s post hoc test were used. A *p*-value of ≤0.05 was considered statistically significant. Principal component analysis (PCA) and cluster analysis (CA) were performed. For cluster analysis, the obtained data for the oil cakes, i.e., dry mass, fat content, acid value, SFA, MUFA, PUFA content, OIT, and TPC, were standardized. Ward’s method was used, and the distances between clusters were estimated using the Euclidean distance.

#### 2.2.2. Methods Used for the Biotransformation Experiments

##### Microorganisms and Growth Media

*Yarrowia lipolytica* KKP379 was obtained from the Collection of Industrial Microorganisms at the Prof. Wacław Dąbrowski Institute of Agricultural and Food Biotechnology (State Research Institute, Warsaw, Poland). The strains *Lactobacillus plantarum*, *Lactobacillus acidophilus*, *Lactobacillus casei*, *Lactobacillus buchneri* 2047, and *Lactobacillus lactis* came from the collection of microbial cultures at the Warsaw University of Life Sciences (Warsaw, Poland). The microorganisms were stored in a −18 °C freezer in 20% (*v*/*v*) glycerol until used.

The growth media used in this work are YPG (for *Yarrowia* species) and MRS (for all *Lactobacillus* species). YPG composition (BTL, Łódź, Poland): yeast extract (10 g/L), peptone (20 g/L), glucose (20 g/L). MRS broth composition (Merc): casein peptone (10 g/L), meat extract (8 g/L), yeast extract (4 g/L), *D*(+)-glucose (20 g/L), dipotassium hydrogen phosphate (2 g/L), Tween^®^ 80 (1 g/L), di-ammonium hydrogen citrate (2 g/L), sodium acetate (5 g/L), magnesium sulfate (0.2 g/L), and manganese sulfate 0.04 (g/L).

##### Screening of Bacteria for Oleic Acid Hydration Capacity

The preculture of lactic acid bacteria was carried out in 40 mL MRS broth in Schott bottles with a total capacity of 100 mL. Bacterial cultures were incubated at 37 °C (Memmert IN 110 incubator) for approximately 24 h to obtain a cell suspension titer of about 10^6^ CFU/mL. After this time, bacterial cells, in the amount of 1% (*v*/*v*), were transplanted onto 50 mL of liquid MRS media and incubated at 37 °C in anaerobic conditions. As soon as the lactic acid bacteria started to grow and were in the log phase (8–10 h), varied concentrations of oleic acid—0.2%, 0.5%, 1%, and 5% (*w*/*v*)—were added. Before introducing it into the medium, oleic acid was dissolved in acetone, and Tween 80 was added (0.1% *v*/*v*) to emulsify the substrate better. To check the progress of oleic acid hydration, after 48 and 96 h of biotransformation, 2 mL samples were taken and acidified to a pH of approx. 3 by adding 0.1 M HCl (0.1 mL) and then extracted with 2 mL ethyl acetate. The organic phase was dried with anhydrous magnesium sulfate, filtered, and then derivatized for GC-MS analysis.

##### Procedure for the Direct Biotransformation of Cake Oil into Lactone Derivatives

The selected strain of lactic acid bacteria was used in a two-pot experiment of biotransformation of oil from oil cakes to lactone derivatives. For this purpose, a preculture of lactic acid bacteria grown as described in Screening of Bacteria for Oleic Acid Hydration Capacity was transplanted into 50 mL of liquid MRS medium, and an anaerobic culture was carried out at 37 °C. After approx. 8 h of cultivation (when fermentation was in the logarithmic growth phase), 1 g of oil extracted from oil cakes or 5 g of oil cakes crushed in a mortar, Tween 80 (0.1% *v*/*v*) and *Candida antarctica* lipase (300 mg/L) were sterile added at once to the medium. Oil hydrolysis and fatty acid hydration were carried out for 48–72 h. After this time, the mixture was centrifuged (MPW-352 centrifuge) for 5 min at 8000 rpm/min, maintaining sterile conditions.

At the same time, a preculture of the yeast *Yarrowia lipolytica* was cultivated. The yeast was grown in Erlenmeyer flasks containing 50 mL of YPG medium at 28 °C for 24 h on an IKA KS 4000 shaker at 140 rpm. After this time, the *Yarrowia* was inoculated into the proper biotransformation culture medium, which consisted of 100 mL of YPG medium, to which the supernatant, obtained after centrifugation of cells from the *Lactobacillus* culture, was added sterilely. Biotransformation was carried out in Erlenmeyer flasks with baffles for 7 days, and samples were taken for chromatographic analyses at specific times.

##### Analytical Methods and Characterization of Substrates and Products from Biotransformation Experiments

GC-MS analysis was used to identify compounds in the post-reaction mixture. Analysis was performed using an HP6890 gas chromatogram with a mass detector and a column HP-5MS (30 m × 0.25 mm × 0.25 µm, Agilent Technologies, Santa Clara, CA, USA). The following temperature program was employed: 60 °C (1 min)—8 °C/min—160 °C—15 °C/min—250 °C (5 min)—25 °C/min—300 °C (10 min); carrier gas was He, a constant flow rate was applied at the level 1 mL/min; and split ratio: 1:30. The samples for GC-MS were incubated with a trimethylsilyldiazomethane solution (Sigma-Aldrich) to derivatize carboxylic acids by transformation into the respective methyl esters.

Methyl 4-ketolaurate: t_r_ 14.06 min

GC-MS (EI): *m*/*z* (%) = 228 (1), 197 (10), 141 (21), 130 (88), 115 (57), 98 (100), 87 (13), 81 (8), 71 (31), 55 (40), 43 (21).

γ-Dodecalactone: t_r_ 14.21 min

GC-MS (EI): *m*/*z* (%) = 198 [M+] (<1), 180 (2), 162 (1), 151 (2), 136 (5), 128 (11), 114 (4), 100 (6), 96 (5), 85 (100), 69 (8), 55 (12), 41 (10).

Oleic acid methyl ester: t_r_ 18.47 min

GC-MS (EI): *m*/*z* (%) = 296 [M^+^] (7), 264 (49), 235 (6), 222 (30), 180 (19), 166 (10), 152 (12), 137 (17), 123 (26), 110 (32), 97 (62), 83 (68), 69 (79), 55 (100).

Methyl 4-ketostearate: tr 20.43 min

GC-MS (EI): *m*/*z* (%) = 312 [M+] (2), 281 (23), 239 (5), 227 (5), 214 (52), 199 (40), 182 (11), 156 (100), 141 (67), 125 (86), 97 (60), 81 (31), 71 (90), 55 (89).

Methyl 10-acetoxystearate: t_r_ 20.74 min

GC-MS (EI): *m*/*z* (%) = 313 [M+ -MeCO] (6), 296 [M+ -AcOH] (3), 281 (17), 264 (31), 243 (11), 222 (9), 201 (100), 169 (64), 157 (16), 125 (21), 97 (18), 83 (19), 69 (21), 55 (27).

## 3. Results and Discussion

The contemporary challenge of science, due to the increasingly visible problems of the planet, is to search for new strategies for achieving sustainable development goals by making the best use of our resources and eliminating waste. This involves developing and optimizing production processes that can process waste in an environmentally friendly and economically sustainable manner. Waste valorization is considered an auspicious approach that should be used with the principles of the circular economy [29,30].

This study evaluates the possibility of using oil cakes—waste products from pressing oil from oil seeds in the biosynthesis of aroma compounds—and fits nicely into the abovementioned trend. The problem of the OleoVital company (Grajów, Poland) with waste disposal prompted us to evaluate these by-products’ quality and attempt their management. Previous experiences with the biotransformation of castor oil to gamma-decalactone and awareness of the limitations of this process [31,32] directed our activities toward biotransformation trials involving non-hydroxylated fatty acids.

### 3.1. Physiochemical Properties of Oil Cakes and Oil Extracted

Five types of oil cakes from hemp seeds, rapeseed, safflower, camelina, and flax were analyzed in terms of dry matter, fat, and protein content. The results of these analyses are summarized in Table 1.

The analysis of the dry matter content in all five OleoVital cakes showed that they contain similar amounts of water. The dry matter was around 85–88% and is slightly lower than in the literature, where the values oscillate between 90 and 93% [33,34]. Protein content ranges from 15.5% to 30.0%. The highest concentration was recorded in flax and rapeseed cakes. The fat content oscillated between 13% and 20%. The highest lipid fraction content was found in rapeseed and camelina cakes. The fat content in these raw materials was around 6–7% higher than in safflower or hemp cakes. The protein and fat content are within the range provided by other authors, 19.4–45.0% for protein and 8.9–31.3% for fat, respectively [33,35,36]. The differences in the composition of oil cakes can vary significantly depending on the quality of the seeds, the extraction technique, and the storage parameters [36].

To select the cakes with the highest potential from the point of view of the two-pot lactones biosynthesis process, in the next phase of the research, the oil extracted from them was analyzed by determining the acid value (AV), oxidative stability, polyphenol content, and fatty acid profile. The physicochemical properties of the oil extracted from the oil cakes are presented in Table 2.

The acid value of oil obtained from the oil cakes ranges from 6.53 mg KOH/g to 35.03 mg KOH/g of oil. The highest AV was characteristic of oil extracted from rapeseed cake—35.03 ± 1.10 mg KOH/g, and the lowest was characteristic of oil from linseed cake—with a value of 6.53 ± 0.91 mg KOH/g. Similar AVs were characteristic of oil from safflower cake (12.59 ± 0.87 mg KOH/g) and camelina (11.39 ± 0.99 mg KOH/g). The acid value, which indicates the hydrolysis process occurring in the oil, resulting in the production of free fatty acids, depends on many factors. The temperature [37], prolonged storage [38], the type of cake, and microbiological contamination [39] can cause higher values of their acid number. Milczarek and Osek, analyzing the lipid fraction of rapeseed cake, showed that it is characterized by an acid value over 10 times higher than oil [40].

The oxidation induction time analyzed by the PDSC technique allowed for determining the stability of the lipid fraction of the oil cakes. OIT in the analyzed samples ranged from 19.92 to 524 min. The lowest oxidation time was recorded for hemp and safflower oil. The oils from flax oil cakes and camelina were more resistant to oxidation. Rapeseed oil was characterized by over 10 times higher stability. Resistance to thermal oxidation is a specific feature of each oil. The fatty acid composition influences it, especially the content of polyunsaturated fatty acids, as well as antioxidants like tocopherols, polyphenols, and prooxidants, for instance, chlorophylls and phospholipids [38].

Total phenolic content is the standard method of assessing antioxidant capacity. The lipid fractions of oil obtained from cakes showed antioxidant activity ranging from 0.17 ± 0.02 to 0.82 ± 0.05 mg GAE/1 g of oil. The lowest TPC values were recorded for the lipid fraction extracted from camelina and rapeseed oil cakes, while the highest were recorded for flax and safflower oil cakes. The obtained results are similar to the values given in the literature. Teh et al. [41] examined the total phenolic content in hemp, flax, and rapeseed press cakes. They showed that the extracted oil contains a much lower antioxidant activity than the raw material. The authors emphasized that the recovery of phenolic compounds and flavonoids from press oil cakes depends on the plant material type and the extraction solvent. The determined total polyphenol content is not correlated with the obtained results of oxidative induction time. This may indicate that OIT mainly depends on the fatty acid composition of the extracted oils.

**Figure 1:**
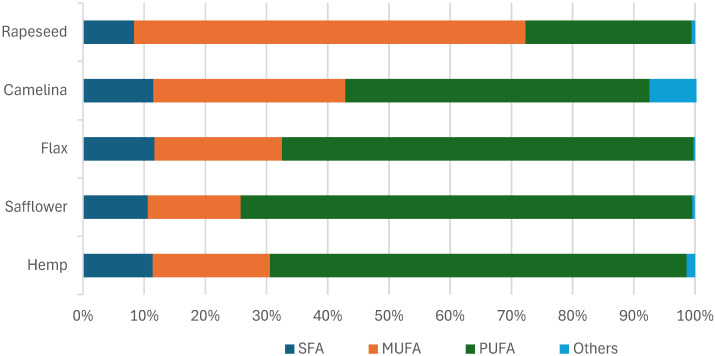
Graph showing the percentage of saturated, monounsaturated, and polyunsaturated acids in oils extracted from five different oil cakes.

The fatty acid profile was analyzed to fully characterize the lipid fraction extracted from individual cakes. The results of chromatographic (GC) determinations, presenting the percentage share of individual acids, are presented in Table 3. Additionally, Figure 1 shows the total share of saturated, monounsaturated, and polyunsaturated fatty acids in the lipid fractions of individual oil cakes.

Analyzing the data from Table 3 and Figure 1, it was observed that in each of the analyzed oils, regardless of their source, monounsaturated and polyunsaturated acids predominate. Hemp cake oil contains mainly polyunsaturated acids (totaling approx. 68%), of which approx. 44.77 ± 0.87% is linoleic acid from the *n*-6 family, and approx. 23.27 ± 0.13% linolenic acid, characteristic of the *n*-3 family. Oleic acid (MUFA) constituted an 18.86 ± 1.02% fraction. Saturated acids, mainly palmitic and stearic, were in the minority (approx. 11%). The results of this analysis are consistent with the data published by Irakli et al. [42], who showed that in the oil pressed from hemp seeds, linoleic and alpha-linolenic acids predominated, constituting approx. 53.4% and 12.1%, respectively. Palmitic acid (7.1% to 9.1%), stearic acid (2.1% to 2.8%), and arachidic acid were identified among the saturated acids. The authors emphasized the favorable ratio of acids belonging to the *n*-6 family, almost twice as much as the *n*-3 family.

The next analyzed oils from safflower and flax cakes were characterized by a similar general fatty acid profile to the previously discussed fractions from hemp. The largest share was also made up of polyunsaturated acids, about 74% in safflower oil and 67% in flax oil. Of the monounsaturated acids, oleic acid was identified at a concentration of about 15.29 ± 0.11% and 20.66 ± 1.02% (for safflower and flax, respectively). Nearly 10% of the lipid fraction in both oils was saturated: palmitic and stearic acids. The results described above were similar to those obtained by other authors [43,44], where it was indicated that flaxseed oil consisted mainly of linolenic acid (47–58%), oleic acid (16–21%), linoleic acid (15–16%), stearic acid (3–7%), and palmitic acid (5–9%), which together constituted about 99% of all fatty acids.

The oil from camelina cake was characterized by a higher share of monounsaturated acids than the oils discussed earlier. The components of this fraction were oleic acid (17.58 ± 1.05%) and eicosenoic acid (13.18 ± 1.22%), with a total content of about 31%. The PUFA fraction did not exceed 50%—alpha-linolenic acid constituted 29.22 ± 0.97%, and linoleic acid 20.43 ± 0.80%. Palmitic acid (5.72 ± 0.54%) and stearic acid (3.00 ± 0.03%) were identified in the composition of saturated acids.

A different fatty acid profile was observed in the case of rapeseed cake oil. Oleic acid MUFA) dominated it at the level of 62.48 ± 1.14%. PUFA acids (linoleic and alpha-linoleic acid) accounted for approx. 27% of the total. Saturated acids did not exceed 8.5%, of which palmitic acid accounted for 5.36 ± 0.78% and stearic acid 2.19 ± 0.09%. The literature [33,45] confirms that rapeseed oil is characterized by a low level (5–7%) of saturated fatty acids (SFA) and significant amounts of monounsaturated fatty acids (MUFA) and polyunsaturated fatty acids (PUFA), including 52–61% of oleic acid, 20–23.5% of linoleic acid, and 9.4–11% of α-linolenic acid, plant sterols (0.53–0.97%), and tocopherols (700–1200 ppm).

### 3.2. Cluster Analysis and PCA Results of Oil Cakes

**Figure 2:**
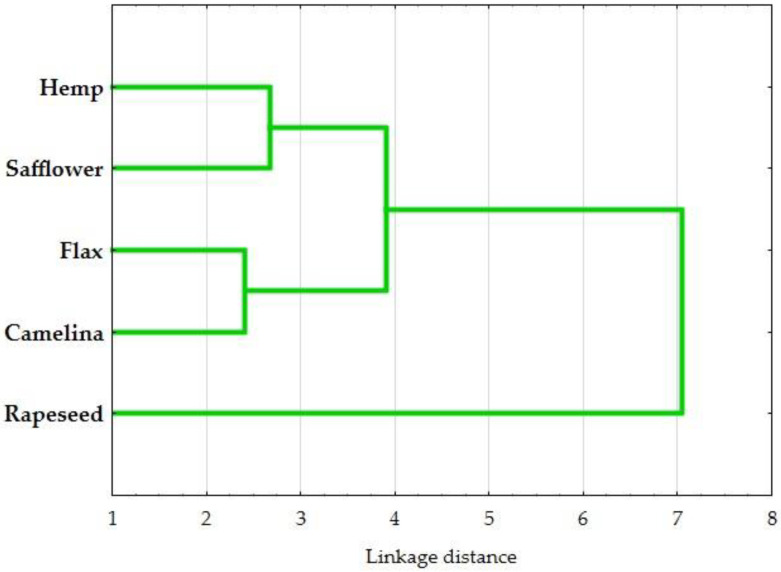
Dendrogram showing results of a hierarchical cluster analysis for oil cakes.

The dendrogram resulting from cluster analysis ordered quality parameters of oil cakes hierarchically, applying the nearest neighbor method with the Euclidean distance measure based on the presence or absence of a particular component. This study classified oil cake samples by the different quality parameters. Figure 2 shows the dendrogram obtained from hierarchical cluster analysis, where two clusters can be identified. Cluster 1 contains rapeseed oil cakes, and cluster 2 contains the remaining oil cakes (hamp, safflower, flax, and camelina).

**Figure 3:**
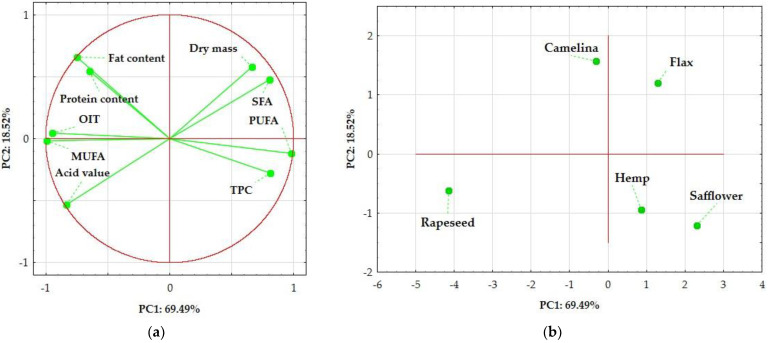
The scores plot of principal component analysis PCA (PC1xPC2) for oil cakes, based on physicochemical parameters of oil cakes and their fatty acid composition: (**a**) PCA loading plot of two principal components; and (**b**) score plot presenting analyzed samples in term of PC1 vs. PC2. Markings: OIT—oxidative induction time; TPC—total phenolic content; SFA—saturated fatty acids; MUFA—monounsaturated fatty acids; and PUFA—polyunsaturated fatty acids.

Principal Component Analysis (PCA) is a statistical technique that extracts and stores the most critical data. PCA creates linear combinations of principal components that describe the variability between individual features. To determine differences and similarities (correlations) between the analyzed oil cakes in terms of the physicochemical properties (oxidative induction time, total phenolic content, and acid value) as well as composition (the content of water, crude protein, fat, and profile of fatty acids), a significant component analysis with classification (PCA) was performed (Figure 3a). The main factors accounted for 88.01% of the variation (PC1: 69.49 and PC2: 18.52%). As shown in the presented biplot (Figure 3b), oil cakes were grouped into three groups based on similarity: camelina and flax, and hemp and safflower were found in the same groups. A different group was formed by rapeseed oil cake.

The analysis of the physicochemical parameters of the cakes and the lipid fraction extracted from them, verified by statistical analysis, allowed us to conclude that the cakes are a valuable waste raw material that can be popular not only in terms of people and animal nutrition [33,46] but also have an enormous potential for biotechnological use. Our idea for the valorization of the cakes was to use the lipid fraction extracted from them in the biotechnological synthesis of aroma compounds from the lactone group. Based on the analysis of physicochemical parameters and, above all, the fatty acid profile of the lipid fraction of the five analyzed types of oil cakes, rapeseed cakes were selected for further studies on the synthesis of gamma-dodecalactone. The choice of this type of cake was dictated by, among others, the most significant amount of lipid fraction in them (approx. 20% of their mass—Table 1), the highest oxidative stability (Table 2), and a fatty acid profile favorable from the point of view of the planned biotransformation, in which oleic acid predominates (over 60%—Table 3). Moreover, due to the significant area of rapeseed cultivation and rapeseed oil production in Poland (the year 2023/2024—3.633 million tons of rapeseed were processed in Poland) [47], rapeseed cakes constitute the essential waste of all analyzed types.

### 3.3. Research on a Two-Pot Process for the Biotransformation of Oil from Oil Cakes into High-Value Natural Lactone

The production of lactones from unhydroxylated fatty acids requires their hydroxylation at the first stage of the process, which takes place via oleate hydratase (EC 4.2.1.53) [48]. Research on the microbiological hydration of unsaturated fatty acids has been conducted since 1960, when oleic acid was successfully hydrated to 10-hydroxystearic acid via the bacteria of the Pseudomonas species [49]. Because among microorganisms, the most promising genus active in the hydration of the double bond of ∆^9−10^ C18 unsaturated fatty acids are the probiotic bacteria Lactobacillus and Bifidobacterium [26], at the initial stage of this study, the effectiveness of selected Lactobacillus species in the hydration of pure oleic acid was analyzed.

#### 3.3.1. Screening of Lactic Acid Bacteria for Oleic Acid Biotransformation

Five strains of probiotic bacteria were selected for a comprehensive analysis of fatty acid hydratase activity: *Lactobacillus buchneri, Lactobacillus acidophilus*, *Lactobacillus casei*, *Lactobacillus plantarum*, and *Lactobacillus lactis.* All the mentioned strains grew in an anaerobic environment because they are facultative anaerobes in an MRS medium at the physiological temperature of 37 °C. In the exponential growth phase of bacteria, oleic acid was introduced at a concentration ranging from 0.2 to 5% (*w*/*v*). The results of this experiment are summarized in Table 4.

Based on the results, it was observed that all analyzed lactic acid bacterial strains possessed oleate hydratase activity. However, their biocatalytic abilities varied depending on the strain and the oleic acid concentration. Among the five *Lactobacillus* strains, the highest activity of oleic acid hydration to 10-hydroxystearic acid was characteristic of the *L. plantarum* strain. The biotransformation efficiency was over 60% (65.7 ± 0.9% after 48 h of reaction and 67.2 ± 0.8% after 96 h). The lowest enzyme activity, lower by half compared to *L. plantarum*, was characteristic of the *Lactobacillus buchneri* strain—26.7 ± 1.1% after 48 h and 29.0 ± 1.4% after 96 h. The remaining microorganisms carried out the biotransformation of oleic acid with an efficiency in the range of 30–40%. Comparing the time of hydration, from the point of view of reaction efficiency, extending the reaction by 2 days did not significantly affect the increase in the concentration of 10-hydroxystearic acid. Extending the action of bacteria from 48 h to 96 h contributed slightly to the rise in the reaction efficiency, which indicates that oleate hydratase enzymes were most active in the first 2 days of biotransformation. While conducting research at different concentrations of oleic acid ranging from 0.2 to 5%, it was observed that the hydration reaction′s efficiency decreased at the highest tested concentration level of 5%, regardless of the bacterial strain used. This may be because, with a higher content of fatty acid in the substrate, bacterial cells multiply less, resulting in a lower concentration of the enzyme and, thus, of the substrate in the post-reaction mixture.

The activity of oleate hydratase in *Lactobacillus* bacteria is confirmed by the results of Serra and De Simeis [25,50], who showed that *Lactobacillus rhamnosus* ATTC 53103 and *Lactobacillus plantarum* 299V are capable of transforming oleic acid into (*R*)-10-hydroxystearic acid, with excellent efficiency and high enantiomeric purity, at the level of ee >95%. In contrast to the results of this article, the authors noticed that with a longer incubation time (80 h), the concentration of (*R*)-10-hydroxystearic acid in the medium increases, formally corresponding to an increased rate of oleic acid transformation. The authors explained this by the fact that 10-hydroxystearic acid has a very low solubility in the biotransformation environment and begins to precipitate immediately after formation. Therefore, the activity of the probiotic strain is not inhibited by it because the constant rate of its formation is maintained. However, the authors used an oleic acid concentration that was more than eight times lower in the reaction than in the experiment we described.

#### 3.3.2. Biotransformation of Oil from Oil Cakes into γ-Dodecalactone

**Figure 4:**
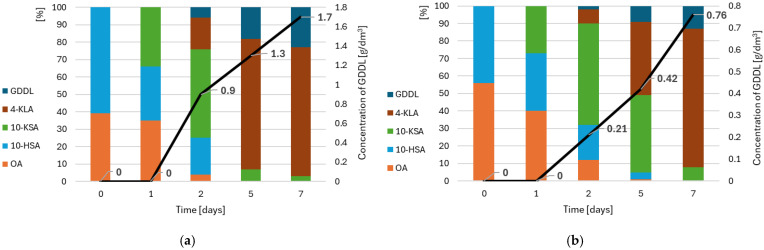
Results of two-pot biotransformation of oil from rapeseed oil cakes carried via lactic acid bacteria *Lactobacillus plantarum* and oleaginous yeast *Yarrowia lipolytica*: (**a**) results from the biotransformation with the participation of extracted oil; and (**b**) results from the biotransformation through the involvement of ground rapeseed oil cakes. Markings: OA—oleic acid; 10-HSA-10-hydroxystearic acid; 10-KSA—10-ketostearaic acid; 4-KLA—4-ketolauric acid; and GDDL—gamma-dodecalactone.

Based on the potential of five selected lactic acid bacteria strains, the one with the highest efficiency of oleic acid biotransformation—*Lactobacillus plantarum*—was chosen for further studies on two-pot lactone biosynthesis. In the next stage of the experiments, a biotransformation reaction was carried out using oil extracted from rapeseed cakes or ground, raw rapeseed oil cakes. Using these substrates, triacylglycerol hydrolysis was necessary, which was carried out by immobilized *Candida antarctica* lipase. After hydrolysis (mediated by lipases) and hydration (using *Lactobacillus plantarum*) carried out under anaerobic conditions, the reaction mixture was centrifuged, and the supernatant was sterilely added to the YPG medium in an aerobic culture of *Yarrowia lipolytica* yeast. The results of this experiment are shown in Figure 4.

The products formed during the biotransformation process, identified by GC–MS analysis, confirm the possibility of synthesis of gamma-dodecalactone from unhydroxylated fatty acid—oleic acid. In the two-pot biotransformation, *Lactobacillus plantarum* was responsible for the hydroxylation of oleic acid to 10-hydroxystearic acid (10-HSA). Regardless of whether the oil was extracted from rapeseed oil cake or ground, raw rapeseed oil cake was used in the reaction, and 10-HSA was present in the reaction mixture after the first stage of biotransformation (in anaerobic conditions) at a concentration of 61% and 44%, respectively (Figure 4—0 day). With the beginning of the second (aerobic) stage of biotransformation with the participation of *Yarrowia* yeast in the reaction mixture, after 24 h of reaction, 10-ketostearic acid (10-KSA) appeared (34% in Figure 4a and 27% in Figure 4b, respectively), a product formed after the oxidation process of 10-HSA. This is probably the effect of the activity of yeast alcohol dehydrogenase. Gatter et al. [51] describe that *Yarrowia lipolytica* has three alcohol dehydrogenase genes, ADH1, ADH2, and ADH3, of which ADH1 and ADH3, in particular, are responsible for the oxidation of long-chain *n*-alkanes and alcohols. The concentration of 10-KSA in the culture with extracted oil was also high on the 3rd day of the reaction (approx. 51% of the composition of the analyzed sample); on the 5th day of biotransformation, it decreased to below 10%. In the case of the culture using ground oil cakes, the concentration of 10-KSA at the level of approx. 44% was still noted on the 5th day of the reaction. On the 7th day, the content of the compound decreased to 7%. The decrease in the concentration of 10-KSA results from its transformation into 4-ketolauric acid (4-KLA). This occurs in the β-oxidation cycle carried out in the peroxisomes of *Yarrowia lopolytica* yeast cells [15]. As a result of three β-oxidation cycles, the chain is shortened by six carbon units, resulting in the formation of 4-ketolauric acid, which, in appropriate conditions (at low pH), cyclizes to gamma-dodecalactone (GDDL). In both experiments (Figure 4a,b), 4-ketolauric acid is identified in samples taken after 2 days of the biotransformation reaction, and its high concentration is observed at the end of the analyzed reaction period (days 5 and 7).

This approach described above, indicating that lactic acid bacteria are responsible for the hydration stage of the unsaturated fatty acid and yeast for its further oxidative degradation leading to the cyclic ester, is confirmed in the literature [52,53,54]. There are also described cases of synthesis of γ-dodecalactone from 10-hydroxystearic acid or fatty acid by yeast strains—*Mortierella isabelline*, *Sporobolomyces odorus*, *Sporidiobolus salmonicolor*, and *Waltomyces lipofer* [55]. In most publications, however, the final concentration of lactone in the biotransformation medium is not specified, and the results are given as efficiency relative to the substrate used. Only a few authors indicated the amount of GDDL they received. Han Ohan [56] describes the possibility of synthesizing 4.1 g/dm^3^ of GDDL from 19.2 g/dm^3^ of dodecanoic acid after 24 h via the fungi *Mortierella isabelline*. Jung-Ung [55] refers to the production of 3.5 g/dm^3^ of γ-dodecalactone from 14.4 g/dm^3^ of 10-hydroxystearic acid after 18 h by *Yarroiwa lipolytica*. In our experiment, approximately 1.7 g/dm^3^ of GDDL was obtained when 20 g/dm^3^ of oil extracted from oil cakes was used in the reaction or 0.76 g/dm^3^ when 100 g/dm^3^ of raw oil cakes were used in the biotransformation. Taking into account the fact that the GDDL concentrations reported in the literature were achieved using substrates directly included in the biotransformation cycle, the lactone concentration results presented in this article can be considered high because oil or raw cakes were used as a substrate which required an additional stage of hydrolysis with the participation of lipases.

## 4. Conclusions

One of the challenges of the modern world is the procedure leading to the reduction in waste generation, which is consistent with the assumptions of the circular economy, which assumes the rational use of resources, with the longest possible circulation of raw materials and waste minimization. In this article, we have developed and presented a new biotechnological process leading to the synthesis of dodecalactone (C_12_H_22_O_2_)—a cyclic ester with a creamy-peach aroma, using waste raw materials such as oil cakes—a by-product of the oil pressing process. The process is based on the biocatalytic activity and selectivity of the strain of the lactic acid bacteria *Lactobacillus plantarum* and the oleaginous yeast *Yarrowia lipolytica* and is carried out using oil extracted from rapeseed oil cakes or raw oil cakes. The complete biotransformation is based on three different biocatalytic steps: hydrolysis of triacylglycerols using a commercial lipase, hydration of oleic acid by lactic acid bacteria (anaerobic process), and beta-oxidation by yeast (aerobic process). The entire transformation takes place in a two-pot process. The microorganisms proposed in the biotransformation are considered safe and have the GRAS status. The proposed biotransformation process yields about 1.7 g/dm^3^ of GDDL after 7 days of reaction when using oil extracted from cakes or almost 0.8 g/dm^3^ when using raw, ground cakes. The obtained efficiency of the process is satisfactory, especially in comparison with other described experiments, in which the proposed lactone synthesis requires isolation of intermediate products from the mixture HFA or uses potentially pathogenic or genetically modified microorganisms. Considering the importance of γ-dodecalactone applications (food and cosmetics industry) and the economic benefits of sustainable management of by-products (waste valorization), the two-pot biotransformation of fatty acids catalyzed by bacteria and yeast is worth optimizing and further investigating.

## Figures and Tables

**Table 1:** Composition of oil cakes.

Oil Cakes	Parameters of Analysis		
	Dry Matter [%]	Crude Protein [%]	Fat [%]
Hemp	85.70 ^a^ ± 2.12	26.02 ^a^ ± 0.51	14.69 ^a^ ± 2.17
Safflower	88.02 ^b^ ± 2.26	15.47 ^b^ ± 0.31	13.82 ^a^ ± 1.56
Flax	88.45 ^b^ ± 0.03	28.80 ^c^ ± 0.82	18.19 ^b^ ± 1.65
Camelina	88.04 ^b^ ± 0.27	27.79 ^ac^ ± 0.42	19.91 ^c^ ± 0.11
Rapeseed	85.31 ^a^ ± 0.12	30.01 ^d^ ± 1.03	20.26 ^c^ ± 1.99

Values reported are mean ± SD (n = 3). Values in columns followed by different superscripts are significantly different (*p* < 0.05).

**Table 2:** The physicochemical properties of oil extracted from oil cakes.

Oil Properties	A Type of Cakes from Which Oil Was Extracted				
	Hemp	Safflower	Flax	Camelina	Rapeseed
Acid value—AV (mg KOH/g)	20.92 ^a^ ± 2.01	12.59 ^b^ ± 0.87	6.53 ^c^ ± 0.91	11.39 ^b^ ± 0.99	35.03 ^d^ ± 1.10
Oxidative induction time—OIT (min)	21.30 ^a^ ± 1.15	19.92 ^a^ ± 0.38	56.95 ^b^ ± 2.21	53.73 ^b^ ± 0.81	524.09 ^c^ ± 12.22
Total phenolic content—TPC (mg GAE/g)	0.62 ^a^ ± 0.08	0.82 ^a^ ± 0.05	0.81 ^a^ ± 0.03	0.17 ^b^ ± 0.02	0.19 ^b^ ± 0.09

Values reported are mean ± SD (n = 3). Values in rows followed by different superscripts are significantly different (*p* < 0.05).

**Table 3:** Fatty acid profile of oil extracted from oil cakes (% of total fatty acids).

Fatty Acid ^1^	Type	Source of Oil Cakes				
		Hemp	Safflower	Flax	Camelina	Rapeseed
C16:0	SFA	6.85 ^a^ ± 0.23	6.65 ^a^ ± 0.04	6.97 ^a^ ± 0.12	5.72 ^a^ ± 0.54	5.36 ^a^ ± 0.78
C18:0	SFA	4.15 ^a^ ± 0.09	3.64 ^a^ ± 0.08	4.53 ^a^ ± 0.08	3.00 ^b^ ± 0.03	2.19 ^b^ ± 0.09
C18:1	MUFA	18.86 ^a^ ± 1.02	15.29 ^b^ ± 0.11	20.66 ^a^ ± 1.02	17.58 ^a^ ± 1.05	62.48 ^c^ ± 1.14
C18:2	PUFA	44.77 ^a^ ± 0.87	42.71 ^a^ ± 1.00	43.07 ^a^ ± 1.11	20.43 ^b^ ± 0.80	20.18 ^b^ ± 0.65
C18:3	PUFA	23.27 ^a^ ± 0.13	31.01 ^b^ ± 0.98	24.11 ^a^ ± 0.89	29.22 ^b^ ± 0.97	6.93 ^c^ ± 0.34
C20:0	SFA	0.40 ^a^ ± 0.02	0.31 ^b^ ± 0.03	0.22 ^c^ ± 0.01	ND	0.81 ^d^ ± 0.03
C20:1	MUFA	0.31 ^a^ ± 0.02	ND	0.18 ^a^ ± 0.02	13.18 ^c^ ± 1.22	1.47 ^d^ ± 0.09
Others		1.39 ^a^ ± 0.05	0.39 ^b^ ± 0.03	0.26 ^b^ ± 0.05	10.87 ^c^ ± 1.42	0.59 ^b^ ± 0.02

^1^ C16:0—palmitic; C18:0—stearic; C18:1—oleic; C18:2—linoleic; C18:3—linolenic; C20:0—eicosanoic; C20:1—eicoseanoic; SFA—saturated; MUFA—monounsaturated; and PUFA—polyunsaturated fatty acids. Values are presented as mean ± standard deviation. Values with different letters are statistically different at *p* < 0.05.

**Table 4:** Results of the microbial biotransformation of oleic acid by lactic acid bacteria.

Microorganism	Biotransformation Time *	Concentration of Oleic Acid (% *w*/*v*)	Yield of (*R*)-10-Hydroxystearic Acid Synthesis (%)
*Lactobacillus buchneri*	48	0.2	26.6 ± 1.4
		0.5	23.3 ± 0.8
		1	26.7 ± 1.1
		5	22.1 ± 0.8
	96	0.2	19.2 ± 0.6
		0.5	22.8 ± 0.6
		1	29.0 ± 1.4
		5	23.3 ± 0.5
*Lactobacillus acidophilus*	48	0.2	45.0 ± 0.7
		0.5	46.2 ± 1.9
		1	48.1 ± 1.5
		5	33.9 ± 0.9
	96	0.2	47.0 ± 02
		0.5	47.5 ± 1.3
		1	49.1 ± 2.5
		5	47.2 ± 1.7
*Lactobacillus casei*	48	0.2	25.2 ± 2.7
		0.5	30.2 ± 0.5
		1	32.5 ± 1.3
		5	24.2 ± 0.6
	96	0.2	27.0 ± 1.2
		0.5	29.9 ± 1.0
		1	33.2 ± 0.2
		5	26.9 ± 2.9
*Lactobacillus plantarum*	48	0.2	41.5 ± 1.3
		0.5	62.0 ± 1.4
		1	65.7 ± 0.9
		5	47.6 ± 1.1
	96	0.2	43.8 ± 2.0
		0.5	62.6 ± 1.8
		1	67.2 ± 0.8
		5	49.1 ± 2.2
*Lactobacillus lactis*	48	0.2	29.8 ± 2.1
		0.5	34.0 ± 1.3
		1	37.8 ± 0.6
		5	39.9 ± 3.5
	96	0.2	37.9 ± 1.1
		0.5	38.3 ± 0.2
		1	40.0 ± 1.5
		5	38.7 ± 0.9

* Number of hours between the addition of the oleic acid and the analysis of the biotransformation medium.

## Data Availability

The original contributions presented in this study are included in the article. Further inquiries can be directed to the corresponding author (J.M.).
